# Genotyping of *Acanthamoeba* From Different Wards of Gonabad Bohlool Hospital, Northeastern Iran

**DOI:** 10.1155/ijm/8672955

**Published:** 2025-08-22

**Authors:** Mitra Salehi, Adel Spotin, Mina Moradi, Morteza Rostamian, Hassan Reza Rokni

**Affiliations:** ^1^Vector-Borne Diseases Research Center, North Khorasan University of Medical Sciences, Bojnourd, Iran; ^2^Immunology Research Center, Tabriz University of Medical Sciences, Tabriz, Iran; ^3^Gonabad Health Center, Gonabad University of Medical Sciences, Gonabad, Iran; ^4^English Department, Faculty of Medicine, Infectious Diseases Research Center, Gonabad University of Medical Sciences, Gonabad, Iran; ^5^Student Research Committee, Gonabad University of Medical Sciences, Gonabad, Iran

**Keywords:** *Acanthamoeba*, genotype, hospital wards, medical equipment

## Abstract

**Background:** *Acanthamoeba* is a free-living amoeba that is widely found in nature in different environments such as soil, water, and dust. This parasite is the cause of amoebic keratitis and granulomatous amoebic encephalitis. This study is aimed at investigating the prevalence and genotypes of *Acanthamoeba* in different wards of Gonabad Bohlool Hospital, northeastern Iran.

**Methods:** One hundred and eighty-three samples were collected from equipment in various wards of Gonabad hospital in 2023; swabs were cultured in nonnutrient agar (NNA) medium with the addition of killed *Escherichia coli*. In positive samples containing stellate cysts, PCR molecular testing was performed using JDP1 and JDP2 primers. To confirm *Acanthamoeba*, genotyping and sequencing were also done.

**Results:** *Acanthamoeba* sp. was observed in 114 out of 183 samples (62.30%). The highest percentage of *Acanthamoeba* contamination was in the emergency ward with 81.82%, and the lowest percentage was in the operating and imaging room with 50%. Moreover, the highest percentage of *Acanthamoeba* contamination was in staff areas and equipment with 66.67%, and the lowest percentage was 56.14% on medical equipment. Also, in this research, the Genotypes T4 (*n* = 10, 43.5%), T3 (*n* = 4, 17.4%), T5 (*n* = 4, 17.4%), T11 (*n* = 3, 13%), and T2 (*n* = 2, 8.7%) were determined.

**Conclusions:** The findings of this study showed that *Acanthamoeba* is more common in the emergency ward and on the surfaces and equipment of the staff areas. Considering the dangerous complications caused by this amoeba, health education to increase awareness in the field of transmission as well as health measures to prevent contamination, including disinfection, is recommended.

## 1. Introduction

Members of the genus *Acanthamoeba* are among the most abundant opportunistic amoebae in nature. *Acanthamoeba* genotypes are found worldwide in fresh water, tap water, swimming pools, ventilators, hospital dialysis units, contact lenses, etc. [1]. *Acanthamoeba* can be seen in two forms: trophozoite and cyst. The size of trophozoites varies between 20 and 45 *μ*m and up to 50 *μ*m depending on the species. The size of the cysts is 13–20 *μ*m and they have a double-layered wall. The inner layer of the cyst (endocyst) may be seen in star, round, oval, or polygonal shapes. *Acanthamoeba* is transmitted to humans through cysts. Cysts, along with water, soil, and dust from outside the body, or from a primary focus in the lungs, nose, and even skin wounds, can enter the body tissues and cause disease [2]. A study in Kashan, examining cancer patients' oral and nasal cavities, found *Acanthamoeba* infection rates of 51% in the oral cavity and 38% in the nasal cavity [3]. So far, 22 different genotypes of *Acanthamoeba* (T1–T22) have been identified. Pathogenic genotypes include T5, T4, and the T4 genotype is most frequently isolated from clinical samples [4]. *Acanthamoeba* causes two important diseases, one of which is granulomatous amoebic encephalitis (GAE), which is seen exclusively in people with immune system defects, including children, people with AIDS and leukemia, and transplant recipients. GAE usually presents with focal neurological deficits and signs of increased intracranial pressure. The prognosis of GAE is poor, and the disease is diagnosed after the death of the patient in most cases [5]. Other important diseases caused by this parasite include painful *Acanthamoeba* keratitis, which leads to blindness. Amoebic keratitis is commonly seen in contact lens wearers and also in immunocompromised individuals [2, 6]. If not treated, *Acanthamoeba* keratitis leads to ulceration and perforation of the stroma, vision loss, and eventually blindness [7]. Studies have shown that *Acanthamoeba* cysts suspended in the air, soil, and pool water are sources of risk for this disease [4]. As mentioned, *Acanthamoeba* is present in various environments such as water sources, soil, and dust. Therefore, humans often deal with this parasite. As a study in America showed, over 80% of the population had antibodies against *Acanthamoeba* [8]. In a study conducted on 55 water samples from 10 recreational rivers around Tehran, it was found that 27.3% of these waters were contaminated with free-living amoebae, 80% of which was related to *Acanthamoeba* [9]. Additionally, a study on 13 dust samples in Iran (IR), a worldwide first, revealed that all isolated genotypes from these sources are considered agents of amoebic keratitis [10]. Similarly, a study in Tehran found that 33.3% of water sources, 45.9% of dust, and 100% of soils were contaminated with *Acanthamoeba*, which confirms the contamination of environmental samples with this parasite [11]. A study in Turkey examined 28 soil samples and 2 water samples. They found 18 samples were positive for *Acanthamoeba* [12]. The presence of *Acanthamoeba* in soil sources is considered one of the important risk factors in disease transmission, especially in susceptible people, which include contact lens users and people with immune system defects. Meanwhile, children are susceptible to *Acanthamoeba* infections due to their lower level of immunity. Since *Acanthamoeba* can be transmitted to humans through contact with soil and dust from outside the body and through the respiratory system and even skin scratches, it is necessary to observe safety precautions [13]. A study showed T4 as the dominant isolated genotype, followed by T5, T3, T2, and T11 [14]. In a study conducted on the dust of Khomein Hospital, 20% were infected with *Acanthamoeba* [15]. Research in IR indicated that out of 1850 water and soil samples, the rate of contamination with *Acanthamoeba* was 42.7% [16]. In a study that was conducted on 122 soil, dust, and water samples in Kashan, 82.8% of the samples were infected with *Acanthamoeba*, and T4, T5, T2, and T7 genotypes were isolated and T4 was the predominant genotype isolated [17]. In a study in Varamin, out of 18 soil samples, 33.3% were infected with *Acanthamoeba*, and all genotypes were determined as T4 [18]. In a study in Tehran, out of 42 samples collected from the hospital's ophthalmology wards, 42.8% were infected with *Acanthamoeba*, of which 92.3% had the T4 genotype [19]. Given the lack of information on *Acanthamoeba* infection in Gonabad and considering the daily influx of patients, some of whom are immunocompromised, this study was aimed at investigating the contamination rate and genotypes of *Acanthamoeba* in various wards of Bohlool Hospital of Gonabad, northeastern IR.

## 2. Materials and Methods

### 2.1. Sampling and Culturing

To investigate potential environmental reservoirs in Bohlool Hospital of Gonabad, we obtained 183 swab samples from equipment across various wards. Sterile swabs were used to collect the samples, which were subsequently cultured on 1.5% nonnutrient agar (NNA). This NNA medium was prepared using Page's Amoeba Saline, a defined saline solution consisting of 2.5 mM NaCl, 1 mM KH_2_PO_4_, 0.5 mM Na_2_HPO_4_, 40 *μ*M CaCl_2_·6H_2_O, and 20 *μ*M MgSO_4_·7H_2_O. The final pH of the solution was adjusted to approximately 6.9 using potassium hydroxide (KOH) [20]. A small scratch was made on the plate so that when the amoeba penetrated the culture medium, it would emerge through the scratch on the surface. After heat-killed *Escherichia coli* was added, the plates were incubated for 1–2 weeks at 28°C–30°C [21].

After preparing NNA medium, a piece of agar containing the highest amoeba density was transferred to it, minimizing fungal contamination. Heat-killed *Escherichia coli was* then added. The culture was subsequently incubated at 30°C for 1 month to facilitate amoeba amplification. Following incubation, both the pelleted material and the supernatant were rigorously washed with sterile phosphate-buffered saline (PBS). *Acanthamoeba* cysts were then collected by centrifugation, and the resulting pellet was retained for subsequent analysis.

### 2.2. DNA Extraction and PCR Amplification Assay

DNA was extracted from positive samples using a phenol–chloroform method, following established protocols [17]. Briefly, cells were lysed using a buffer containing 50 mM NaCl, 10 mM EDTA, and 50 mM Tris-HCl (pH 8.0), supplemented with proteinase K (0.25 mg/mL), and incubated at 56°C overnight. *Acanthamoeba* DNA was then amplified by polymerase chain reaction (PCR) using the JDP primer set (Takapouzist Company, IR): JDP1 forward (5⁣′-GGCCCAGATCGTTTACCGTGAA-3⁣′) and JDP2 reverse (5⁣′-TCTCACAAGCTGCTAGGGGAGTCA-3⁣′). These primers amplify a target region of approximately 500 base pairs. Each 30-*μ*L PCR reaction contained 25 *μ*L of Ampliqon Taq DNA Polymerase Master Mix RED (Denmark), 10 ng of template DNA, and 0.1 *μ*M of each primer. The PCR cycling conditions were as follows: initial denaturation at 94°C for 3 min, followed by 33 cycles of denaturation at 95°C for 35 s, annealing at 56°C for 45 s, and extension at 72°C for 1 min. A final extension step was performed at 72°C for 5 min. Positive and negative controls were included in each PCR run to ensure assay validity. The presence of a 500-bp band upon gel electrophoresis was considered indicative of *Acanthamoeba* DNA, and subsequent sequencing was performed to determine the genotype.

### 2.3. Sequencing, Phylogenetic Analysis, and Haplotype Network

Twenty-three PCR amplicons of the 18S rRNA gene were subjected to Sanger sequencing by Takapouzist Company. Resulting sequences were trimmed, edited, and aligned against reference sequences representing known *Acanthamoeba* genotypes using Sequencher Tmv4.1.4 software. To visualize intraspecific phylogenetic relationships, a haplotype network was constructed using the Median-Joining algorithm implemented in PopART software. Phylogenetic relationships among *Acanthamoeba* isolates were further investigated by constructing a maximum likelihood phylogenetic tree using MEGA v5.05 software. The robustness of the tree topology was assessed by bootstrap analysis with 1000 replicates; nodes with bootstrap support values ≥ 60% were considered well supported. Genetic distances are displayed on the tree using a scale of 0.002 substitutions per site. *Acanthamoeba* T5 genotypes were used as an outgroup to root the tree.

## 3. Results

**Figure 1:**
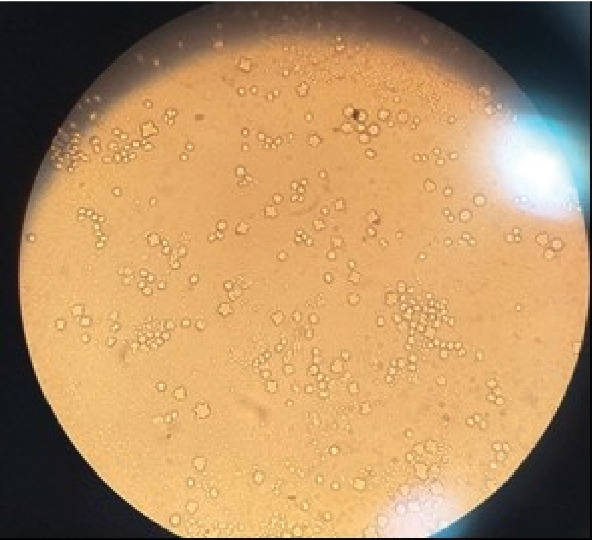
Presence of *Acanthamoeba* cyst in nonnutritive agar culture medium (40× magnification).

*Acanthamoeba* stellate cysts were seen in 114 out of the samples (62.30%) (Table 1 and Figure 1). The highest percentage of *Acanthamoeba* contamination was in the emergency wards with 81.82%, and the lowest percentage was in the operating and imaging room with 50.00% (Table 2). Also, the highest percentage of *Acanthamoeba* contamination was found in staff areas and equipment with 66.67%, and the lowest percentage was in medical devices with 56.14% (Table 1). Also, in this research, the genotypes of T4, T5, T3, T2, and T11 were determined (Table 2).

The result of the chi-square test showed that there was no statistically significant difference in the frequency of *Acanthamoeba* according to levels and equipment (*p* = 0.492) (Table 1). The highest percentage of *Acanthamoeba* contamination was in surfaces and equipment in the staff areas (e.g., nursing desks and stations) with 66.67% and the lowest in medical devices with 56.14% (Table 1).

The result of the chi-square test showed that there was no statistically significant difference in the frequency of *Acanthamoeba* according to the sections (*p* = 0.257). The highest percentage of *Acanthamoeba* contamination was in the emergency wards with 81.82% and the lowest in the operating and imaging room with 50.00%.

**Figure 2:**
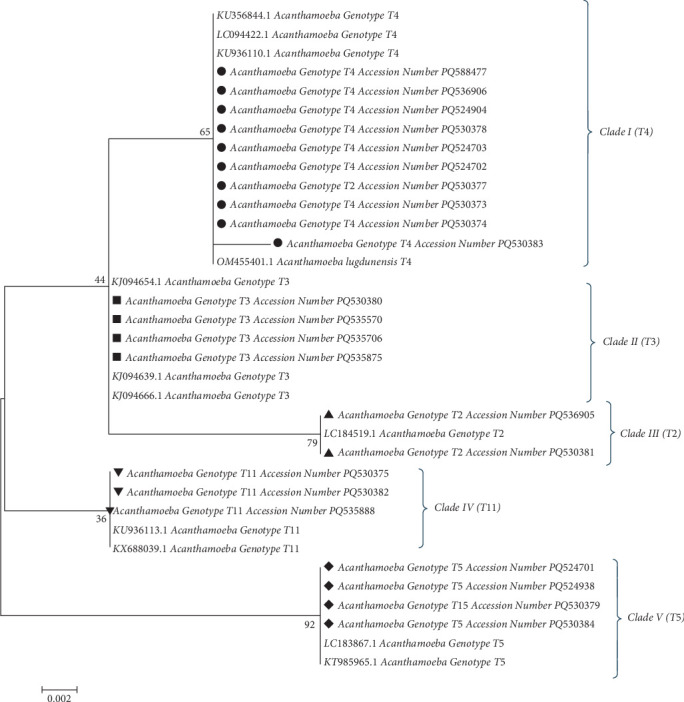
The phylogenetic tree of *Acanthamoeba* genotypes based on the 18S rRNA sequences. The tree was generated using the maximum likelihood algorithm and Kimura 2-parameter mode. The sequences are shown by geometric shapes. The topology of the phylogenetic tree indicated that *Acanthamoeba* genotypes have been classified into five distinct clades, including Clades I (T4), II (T3), III (T2), IV (T11), and V (T5).

Based on sequence analyses of 18S rRNA, five *Acanthamoeba* genotypes were identified, including T4 (*n* = 10, 43.5%), T3 (*n* = 4, 17.4%), T5 (*n* = 4, 17.4%), T11 (*n* = 3, 13%), and T2 (*n* = 2, 8.7%). The topology of the phylogenetic tree indicated that the identified *Acanthamoeba* genotypes have been classified into five distinct clades (Clades I [Genotype T4], II [Genotype T3], III [Genotype T2], IV [Genotype T11], and V [Genotype T5]) compared to other geographical regions of the world. The submitted accession numbers of *Acanthamoeba* genotypes are marked with an asterisk symbol (∗) in Figure 2.

**Figure 3:**
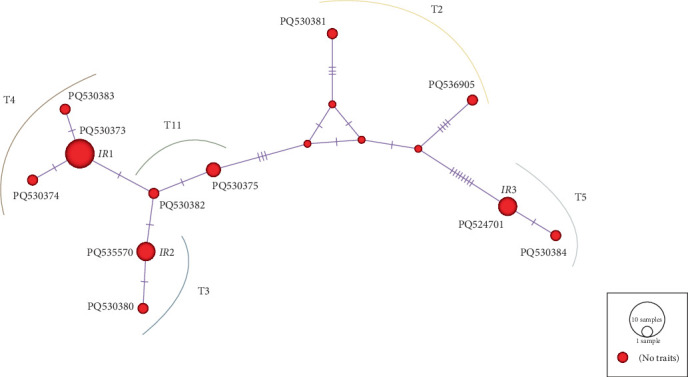
Median-Joining haplotype network of *Acanthamoeba* 18S rRNA genotypes obtained from the current study. IR1 (T4, 30%), IR2 (T3, 13%), and IR3 (T5, 13%) were considered as common haplotypes. The red circles as hypothetical haplotypes are relative to the frequency of each haplotype. Each line between haplotypes indicates a single mutational step.

A statistical parsimony of the sequence haplotypes of 18S rRNA displayed a star-like feature in the identified isolates in northeastern IR including common haplotypes IR1 (T4, 30%), IR2 (T3, 13%), and IR3 (T5, 13%) (Figure 3).

## 4. Discussion

The occurrence of *Acanthamoeba* in the hospital environment may pose a health risk to patients because these organisms can cause severe opportunistic diseases such as keratitis and can also harbor pathogens. This research was carried out to determine the prevalence and genotyping of *Acanthamoeba* in different wards of the hospital in Gonabad in northeastern IR. This parasite is potentially pathogenic in immunocompromised or traumatized individuals and may act as an opportunistic pathogen [5]. In addition, *Acanthamoeba* can be a carrier of some bacteria, viruses, and fungi [22–24]. In this study, the rate of *Acanthamoeba* in the whole sample was 62.30%. The highest percentage of *Acanthamoeba* contamination was in the emergency wards with 81.82%, and the lowest percentage was in the operating and imaging room with 50.00%. Additionally, the highest percentage of *Acanthamoeba* contamination was found in staff areas and equipment (66.67%) and the lowest (56.14%) in medical devices. Few studies were found on the presence of free amoebae in hospital dust in IR and the world. A study in Gilan hospitals found *Acanthamoeba* presence at 69.36%. The frequency of *Acanthamoeba* in chemotherapy, hematology, and eye/ear wards was 38.8%, 29.6%, and 39% (pharynx and nose), respectively, with isolated Genotypes T2, T4, and T11 [25]. In a study in Kashan, a PCR test showed that the rate of *Acanthamoeba* contamination in soil, hospital dust, and stagnant water samples was 62.5%, 52.5%, and 50%, respectively, and T4, T5, T2, T7, and T11 genotypes were isolated, and T4 was the predominant genotype [17]. In a study in five hospitals in Tehran, a total of 70 dust and biofilm samples were collected from the wards of transplantation, pediatrics (malignancies), HIV, leukemia, and oncology and were examined for the presence of FLA using culture and molecular methods. *Acanthamoeba* was detected in 52.9% of immunocompromised patients (organ transplant, HIV, malignancy, leukemia, and oncology) and 42.86% of dust samples in Tehran hospital wards. The overall prevalence of *Acanthamoeba* in these studies was similar to our study, which could indicate the high prevalence of *Acanthamoeba* in the dust of hospital environments, and the predominant genotype was T4 [19, 26]. In a study in Khomein that examined the prevalence of *Acanthamoeba* in hospital dust samples, out of 100 samples, 20 samples were positive, and it was determined that the prevalence of *Acanthamoeba* was 10% in the internal ward, 5% in the emergency ward, and 5% in the eye ward [15]. In a study that was aimed at investigating the extent of *Acanthamoeba* contamination in hemodialysis and dental units in Alexandria, Egypt, 70 samples from these systems were aseptically collected and cultured on NNA at room temperature, followed by morphological confirmation of *Acanthamoeba*. It was done using trichrome-stained smears. This study showed that 42.9% of water samples from hydraulic hemodialysis systems and dental units were positive for *Acanthamoeba*, without statistically significant differences between the two types of units or between samples before and after disinfection for each type of unit. The surgical category of dental clinics had the highest level of contamination (72.7%), while no contamination was observed in the water samples of pediatric dental clinics [27]. In Silvia's study in Brazil, *Acanthamoeba* was observed in 45.75% of dust [28].

In another study, the prevalence of *Acanthamoeba* was found to be 43% on hospital environmental samples in Brazil [29]. In 2007, Carlo et al. reported the presence of *Acanthamoeba* in 35% of dust collected from hospitals in Brazil [30]. The prevalence of *Acanthamoeba* in dust collected from hospitals in Brazil was 34%, 23%, and 35%, and Genotypes T3, T4, and T5 were determined [28–30]. The rate of *Acanthamoeba* contamination in the mentioned studies was lower than the present study, which could be due to the difference in the region and the level of compliance with the disinfection standards of the surfaces. By studying 80 samples from different environmental sources, Rezaian and colleagues in Tehran identified 46.25% of *Acanthamoeba* and found that 100% of the five soil samples were contaminated with *Acanthamoeba*. Also, out of 61 samples collected from environmental dust, 45.9% were positive for *Acanthamoeba* [11]. In a study conducted in Tehran parks, out of 52 soil samples, 14 isolates (26.9%) of *Acanthamoeba* were identified in terms of morphology in the culture medium, and the genotype of all samples was T4 [31]. According to the findings of the study, 33.3% of the soil of Varamin was positive for the presence of *Acanthamoeba* by culture method, and the determined genotype is T4 [18]. In a study conducted in the waters of Kermanshah, 76.66% of the waters were contaminated with *Acanthamoeba*, and Genotypes T4, T2, T5, and T11 were isolated, which is in accordance with the findings of our study [32]. In Shiraz, out of 82 samples, 46 samples had *Acanthamoeba*, and T5 and T15 genotypes were isolated and T4 was the predominant genotype [33]. In a study in Gonabad, it was found that 77% of water, 60% of soil, and 42% of dust were contaminated with *Acanthamoeba*, and the dominant genotype was T4, which is consistent with the results of this research [34]. In this study, we isolated *Acanthamoeba* from different wards of the hospital and isolated Genotypes T4, T5, T3, and T11, which is in accordance with previous studies [25–27], and also, the emergency wards had the highest contamination, which is due to the higher traffic of people. Further, due to its special conditions, it shows that health precautions should be observed more in the emergency wards.

## 5. Conclusion

In this study, the rate of contamination with *Acanthamoeba* in different hospital departments was reported to be 62.30%, which indicates that all hospital departments and equipment should be disinfected more carefully and that adherence to hygiene tips is recommended, especially for immunocompromised patients.

## Figures and Tables

**Table 1:** Distribution of *Acanthamoeba* on surfaces and equipment in different wards of Gonabad Bohlool Hospital.

**Names of surfaces and equipment**	***Acanthamoeba*-positive cases** **N** **(%)**	***Acanthamoeba-*negative cases** **N** **(%)**	**Chi-square test result**
Sick room	50 (64.10)	28 (35.90)	*p* = 0.492
Staff areas	32 (66.67)	16 (33.33)	
Medical devices	32 (56.14)	25 (43.86)	
Total	114 (62.30)	69 (37.70)	

**Table 2:** Distribution of *Acanthamoeba* and its genotype in different wards of Gonabad Bohlool Hospital.

**Ward name**	***Acanthamoeba*-positive cases, ** **N** **(%)**	**Genotype**	***Acanthamoeba-*negative cases, ** **N** **(%)**	**Chi-square test result**
Staff areas	8 (50)	T4	8 (50)	*p* = 0.257
Emergency	18 (81.82)	T2, T4, T4	4 (18.18)	
Children's wards	12 (57.14)	T3, T11, T4	9 (42.86)	
Imaging	9 (50)	T4	9 (50)	
Surgery	12 (63.16)	T5, T3, T4	7 (36.84)	
Internal	14 (77.78)	T3, T11, T4	4 (22.22)	
Dialysis	12 (63.16)	T2, T5, T4	7 (36.84)	
Gynecology and obstetrics	11 (47.83)	T3, T4, T5	12 (52.17)	
Special care	18 (66.67)	T5, T11, T4	9 (33.33)	
Total	114 (62.30)		69 (37.70)	

## Data Availability

Data are available upon request from the corresponding author.
